# Newtonian-cultural motion method: a transdisciplinary method for migrant studies

**DOI:** 10.3389/fsoc.2025.1689957

**Published:** 2025-10-24

**Authors:** M. A. Pavithra, M. Subbulakshmi

**Affiliations:** Vellore Institute of Technology, School of Social Sciences and Languages, Chennai, Tamil Nadu, India

**Keywords:** migration, culture, identity, dominant, residual, emergent, force, motion

## Abstract

This paper introduces a transdisciplinary methodological approach, the Newtonian-Cultural Motion Method (NCMM), which integrates Newton’s laws of motion with Williams’ cultural theory of dominant, residual, and emergent culture. By reconceptualizing identity as motion and culture as force, NCMM offers a dynamic lens for analyzing cultural negotiation in migration narratives. Integrating physics and cultural studies, this method offers a new dimension to rethink identity formation shaped by sociopolitical pressures and inherited cultural inertia. This method is demonstrated through a critical analysis of the diasporic novel *Pachinko* by Min Jin Lee, a multigenerational family saga that traces the experiences of Korean immigrants in Japan. NCMM, explicate how cultural pressure, resistance, and transformation operate across generations of migrant communities. It enables researchers to understand migrant identity as a dynamic process shaped by both historically rooted cultural mass and sociopolitical forces. This new methodological approach provides a basis for the exploration of cultural negotiation, the mechanics of pressure, resistance, and transformation in migrant narratives. By bridging Newton’s law of motion with Williams’ cultural theory, NCMM provides a new transdisciplinary approach in analyzing the dynamics of identity, and transitions of culture. This method refigures identity as motion, and culture as a force.

## 1 Introduction

“Cultural identity is an individual’s sense of self derived from formal or informal membership in groups that transmit and inculcate knowledge, beliefs, values, attitudes, traditions, and ways of life” (Jameson, 2007, p. 199). This understanding of cultural identity is significant in the context of diaspora. “The term diaspora refers today not only to such classic groups as Jews, Greeks, and Armenians, but too much wider categories which reflect processes of politically motivated uprooting and moving of populations, voluntary migration, global communications and transport” (Shuval, 2000, p. 42). The identity of the diasporic individuals living outside their homeland is continually shaped to maintain, negotiate and transform their sense of self and cultural belonging. Migrants’ identity is neither rigid nor monolithic, rather it develops through the continuous process of assimilation, resistance, preservation of cultural roots and emergence of new identities. Immigrants in host land often undergo systemic discrimination and social stigmatization by the dominant society based on their ethnicity, language, and nationality. “Discrimination toward immigrants is evident when immigrants receive differential treatment compared to native-born individuals in a particular domain” (Acolin et al., 2016, as cited in Esses, 2020, p. 511). Immigrants’ identity is multifaceted, as they face challenges in balancing the foreign cultures and preserving their own cultural lineage.

Identity, in this context is regarded as the negotiated outcomes of personal conditions, interpersonal relationships, social environments and institutional structures (La Barbera, 2015). Dhar and Raman (2024) states, “The concept of diaspora identity is a complex and intricate tapestry that is crafted from the threads of dual heritage, the retention of culture, and the perpetual negotiation of belonging in a world that is both shrinking and expanding at the same time” (p. 256). These identity crises and cultural conflicts experienced by migrants are the central features of diasporic life. These challenges offer a theoretical inquiry, for understanding cultural forces and external pressures acting upon migrant subjectivities across time and generations. The identity crises and cultural difficulties are prevalent in the life of immigrants. The external pressures experienced by the migrants are often combined with internal attachments to cultural heritage causing psychological strain or intergenerational fragmentation. Traditional approaches to analyze identity transitions and formation have been inadequate in exploring the relationship between cultural retention and forced assimilation experienced by migrant communities.

To address this theoretical and methodological gap, the present study introduces the Newtonian- Cultural Motion Method (NCMM), a transdisciplinary methodological approach that integrates Newton’s (1687) Law of Motion and Raymond Williams’ (1977) cultural theory of dominant, residual, and emergent culture. To ground this method in existing academic discourse, the literature review section provides an overview of the scholarly contributions. It focuses on migrant identity, cultural hybridity, and interdisciplinary cultural studies. It also highlights the gap in understanding identity shifts through the interpretive lens to examine the cultural shift under pressure. The theoretical framework section details the principles of Newton’ s law of motion and Raymond Williams’ cultural theory on dominant, residual and emergent, and it explains the approach of NCMM toward the migrant studies.

The methodology section introduces and explains NCMM as the transdisciplinary methodological approach for mapping cultural inertia, resistance, and reaction of the migrants and it also justifies its application to literary narratives as sociocultural case studies. The analysis is structured around three laws of motion and three kinds of culture. First, Newton’ s first law of motion (inertia) is merged with dominant culture, emphasizing the rigid nature of host societies that resist the influence and integration of migrant communities. Second, Newton’ s second law of motion is integrated with residual culture, representing the deep attachments of migrant communities toward their homeland and their resistance toward the assimilation into dominant cultural norms. Third, Newton’s third law of motion is paired with the emergent culture interpreting identity formation as a counterforce arising from discrimination and identity negotiation. This tripartite methodological approach allows for a nuanced understanding of migrant identity not as a fixed state, but as a field of motion.

## 2 Review of literature

“Migration is a common human undertaking that is often driven by globalization, politics, and economics” (Shamase and Sekaja, 2025). Analysis on migrants, identity, and culture has been examined widely. The existing literature has explored the diasporic experience and its disrupted notions of culture, selfhood and belonging. Chambers (1994) contributes to the diasporic studies by examining the cultural diversity and its impact on identity and spatial belonging. He highlights the Western beliefs in linear progress by exploring the homelessness of migrants. The above-mentioned research paper is valuable; however, it is discursive in nature but it lacks systematic approach to trace migrants’ long term identity transformation. Werbner (2005), in her study on Pakistani migration to Britain, uses British Muslim diasporic struggle as an example to explore a historical perspective on multicultural citizenship. She examines changing and the negotiated forms of multicultural citizenship rather than those fixed by universal principles. Literary analysis has added depth to the exploration of migration communities and to study their evolving identities. Chemnchu et al. (2025) by analyzing the characters of V. S. Naipaul’s *The Mimic Men* and Caryl Phillips’ *Cambridge,* explains the quest for integration into British society. This process of integration gives rise to complex and new modes of identity, challenging cultural changes in the Western world. These research papers highly concentrate on the thematic interpretation of identity evolution and it lacks structural methodological approach for migrant studies. Drawing on Edward Said’s “Traveling Concepts” theory Saxena and Sharma (2018) analyzes the reconfiguration of migrant identity among ethnic minorities in Britain and the U. S. through postcolonial narratives. Cohen and Kassan (2018) contribute to this discourse from a psychological perspective by developing a qualitative model to explore cultural identity negotiation among emerging adult immigrants. Their findings illuminate the social and psychological factors shaping the young immigrant’s dual cultural identity with a practical implication of counseling psychology. These researches are more conceptual, highlighting the multi-faceted dimension of the migrants particularly the negotiated and the hybrid identity, but it lacks mapping them over time. Migration analysis has traditionally been explored as a construct shaped by cultural and identity negotiation, while traversing geopolitical, linguistic, and cultural borders. Classical diaspora scholarship foregrounds factors such as collective memory, homeland attachment, and host-society discrimination (Safran, 1991; Brah) whereas more recent work in transnationalism highlights the fluid, multi-sited nature of migrant belonging (Vertovec, 1999). In contemporary research, migrants’ multiple belongings and social connectivity is highlighted as a means of transnationalism (Nowicka, 2020). Research applying intersectionality theory underlines the overlapping social identities of migrants. Dhoest (2019) examines the pressures faced by the non-heterosexual women in Belgium navigating religious and gender norms while being distant from family communities. Despite the means of ethnographic and literary analyzes, researchers still lack a concise conceptual framework to map the accumulation of cultural pressures and to catalyze the cultural evolution.

Despite valuable contributions to the study of migrant identity, the existing literature tends to rely on symbolic and thematic analysis which significantly lacks a structured framework. This absence of structural conceptualization confines the understanding of evolution of identities across generations. While ethnographic and literary analysis offer a rich insight, they lack a systematic approach for mapping the influence of cultural pressures over time. The Newtonian—Cultural Motion Method, a transdisciplinary methodological approach fills this gap by conceptualizing identity as motion and culture as force for mapping identity shifts in response to sociopolitical and cultural pressures faced by the migrants.

## 3 Theoretical framework

Raymond Williams’ theory of dominant, residual, and emergent culture provides strong insights in exploring the cultural transitions. Williams proposes the prevailing ideological norms as dominant, the lingering traditions as residual and the emerging alternatives as emergent culture. This existing theory is used by the scholars in literary and media analysis; their use is often metaphorical. In contrast, the NCMM provides a nuanced understanding of forces that drive the mechanisms of identity transformation in migration narratives.

Newton’s three laws of motion: Inertia, Force, and Reciprocal action theorize the process of transformation. It describes how an object’s velocity changes when forces act upon it, how acceleration is proportional to applied force and inversely proportional to mass, and how every action elicits an equal and opposite reaction. When it is interlinked to the cultural sphere, mass could be related to the weight of inherited norms, while force denotes sociopolitical pressures such as assimilation, law, employment, economic or xenophobic discourse. Bourdieu (1990), the French sociologist has previously drawn on physical metaphors, merging social behavior to embodied momentum with his concept known as “habitus”. “Habitus” (Bourdieu, 1977) internalizes social behavior by guiding perception, thought and action. “It is not a “structure” but a durable set of dispositions that are formed, stored, recorded and exert influence to mold forms of human behavior. It may vary in accordance with the social environment, because unstable social domains may produce unstable systems of dispositions that generate irregular patterns of action” (Navarro, 2006, p. 16). Similarly, structuration theory emphasizes that “Structure must not be conceptualized as simply placing constraints upon human agency, but as enabling” (Giddens, 1990, p. 169). Structuration is the recursive interplay between agency and structure.

The sociological frameworks “habitus” and “structuration” highlight the shaping of dispositions and mediation of action by socialization, but they do not systematically analyze the external pressures as the directional, force-driven cultural vectors in migrant identity transformation. The use of physics-based metaphors in the Newtonian–Cultural Motion Method (NCMM) conceptualizes the external pressures as directional cultural vectors with measurable magnitude, velocity, and resistance.

The Newtonian–Cultural Motion Method (NCMM) addresses this gap by treating identity formation as motion through a cultural field structured by dominant, residual, and emergent forces. NCMM provides an analytical lens to explore Newtonian mechanics which supplies the language of vectors, acceleration, and resistance; Williams’ theory furnishes the socio-historical taxonomy against which those vectors are plotted. This hybrid method allows researchers to observe cultural positions of the migrants, their paths of transformation under specific conditions.

To demonstrate its utility, NCMM is used to analyze Min Jin Lee’s *Pachinko* (Lee, 2017), a multi-generational novel chronicling Korean migration to Japan. The text foregrounds recurring moments of cultural inertia, Confucian family ethics, linguistic barriers and sudden surges of force, from colonial oppression to post-war economic shifts. By exhibiting these narrative events as Newtonian interactions within Williams’ cultural divisions, NCMM reveals patterns of pressure, resistance, and transformation that remain opaque under conventional close reading. Ultimately, the method equips the scholars, literary critics, and cultural theorists with a reproducible framework for visualizing identity as a dynamic process governed simultaneously by historically rooted cultural mass and contemporary sociopolitical force.

## 4 Methods details

### 4.1 Raymond Williams’ theory in analyzing migrant culture in fiction

Raymond Williams’ cultural theory, particularly his classification of Culture as dominant, residual, and emergent culture, serves as the most significant tool for understanding the complexity of cultural change. According to Williams (1977), “The complexity of a culture is to be found not only on its variable processes and their social definitions—traditions, institutions, and formations—but also in the dynamic interrelations, at every point in the process of historically varied and variable elements” (p. 120). Dominating culture refers to the dominating customs, beliefs, cultural practices that influence certain communities of people living in the host country. “The residual, by definition, has been effectively formed in the past, but is still active in the cultural process” (p. 122). The residual culture for immigrants is the culture that they bring with them from their homeland to the dominant society in which they live. Emergent culture comprises new cultures, values and practices. Raymond Williams explains emergent culture as, “By ‘emergent’ I mean, first, that new meanings and values, new practices, new relationships and kinds of relationships are continually being created” (p. 122).

In migration studies and literary analysis, this theory allows researchers to identify the inheritance of cultural norms carried into new geopolitical spaces, their interaction with the host land’s dominant culture, and the emergence of new cultural forms in this process. Storey (2009) and Hall (1990) have extended Williams’ ideas into the realm of media and identity studies, emphasizing “identity” often shaped through negotiations between these three cultural forces. However, one significant limitation of Williams’ cultural theory is that it is descriptive rather than dynamic. While it provides a rich methodology for exploring cultural coexistence and contestation, it does not offer tools for examining “how” identities shift, “when” they do so, and under “what” kind of pressures. This method addresses this gap by introducing Newton’s laws of motion as a structural and dynamic method for identity change.

### 4.2 Newton’s laws of motion as cultural mechanics

Sir Isaac Newton’s three laws of motion, describe how physical objects react under forces. These laws include:

- *First Law (Inertia)*: Objects remain in their current state of rest or in uniform motion unless acted upon by an external force.
- *Second Law (F = ma)*: Acceleration is proportional to the force applied and inversely proportional to the mass of an object.
- *Third Law (Action-Reaction)*: Every action has an equal and opposite reaction.

In NCMM, these laws of motion are metaphorically applied to understand the motion of identities in socio-cultural contexts. According to Newton, an object’s path could be altered by an external force. Similarly, a person’s cultural identity is shaped, particularly in diasporic or migratory contexts, where it is resisted or redirected by societal norms, political conditions, and historical legacies. In this method Dominant culture acts as a force of inertia, which makes individuals to remain within prevailing social norms. Residual cultural traditions act as internal mass, which resists sudden change. Emergent culture results from counter-forces, especially from dominant ideologies, that produce alternative new ideologies. NCMM provides a more structured and analytically precise method to map the identity change.

### 4.3 Newtonian-cultural motion method

Newtonian-Cultural Motion Method integrates Raymond Williams’ cultural theory on dominant, residual, and emergent culture and Newton’s law of motion to examine and reconceptualize migrant identity as a form of culture and cultural motion, as a system of interacting forces. The analysis of this methodology as follows:

Cultural Taxonomy (Williams): Cultural theory that is dominant, residual, and emergent cultural forms offer a historical and ideological examination of cultural transitions.

Cultural Dynamics (Newton): By applying Newton’s three laws of motion, it helps to understand identity as a means of movement, how it is maintained through cultural inertia (first law of motion), altered by socio-political acceleration (second law of motion), or reshaped through resistance or reciprocal interactions (third law of motion).

The conceptualization of NCMM provides a structural framework which analyses cultural transitions of migrants by integrating cultural studies with physics metaphorization such as motion, mass and force. This methodological tool enables researchers to map the culture of migrants as a dynamic flow. This physics based cultural analysis offers an unambiguous systematic tool to trace migrants’ cultural identity in terms of intensity, magnitude and direction of cultural pressures over time.

## 5 NCMM: interpreting Newton’s Laws through cultural motion

### 5.1 Newton’s first law of motion (inertia) and dominant culture

Newton’s First Law states that an object remains in its current state unless acted upon by an external force. In terms of cultural context, this law is associated with dominant culture, in which it operates as a force of inertia. Dominant culture comprises prevailing ideologies, institutional practices, and accepted societal behaviors by the dominant society that maintain to continue in social systems. Thus, the tendency of dominant norms persists over time unless disrupted by external influences like a change in legal systems is referred to as Cultural Inertia.

#### 5.1.1 Institutionalized norms and the mechanics of cultural inertia

Cultural inertia operates through the institutionalization of dominant norms. In the book, *The Social Construction of Reality,* Berger and Luckmann (1966) explain, “Institutionalization occurs whenever there is a reciprocal typification of habitualized actions by types of actors. Put differently, any such typification is an institution” (p. 72). This suggests that, when individuals are repeatedly subjected to certain practices that become socially legitimatized, those practices eventually become institutionalized. Within dominant culture the existing cultural norms, legal, educational, religious practices are constantly followed by the citizens of a particular land or country and it does automatically become institutionalized. This institutionalization forms a framework within which the dominant society operates, making it difficult for migrants to penetrate into these existing structural forms.

#### 5.1.2 Migrant identity as dynamic force against inertial dominance

Migrants not only have their individual identities but also, they carry collective histories, cultural traditions with them. These factors contrast with, and often challenge the dominant cultural norms. Migrants try to enter as a dynamic force; however, dominant culture resists this motion. Here, Newton’s first law of motion becomes a metaphor for the socio-cultural dynamics between the force of migrant identity and the inertia of the dominant culture. The host culture maintains its strict norms unless external forces like legalization is applied. This inertia is not passive but deeply entrenched. Gramsci (1971) describes this as “hegemony,” “it means a political leadership based on the consent of the led, a consent which is secured by the diffusion and popularization of the world view of the ruling class” (Bates, 1975, p. 352). Within this hegemonic framework the cultural inertia of dominant culture presents its values, ideologies, languages, and social structures as natural and universal. Thus, migrant cultural belongings are considered inferior by the dominant cultural society. This discrimination creates a huge impact on the psychological wellbeing of the migrants. “If the individual feels isolated from his or her culture, unaccepted by the “majority culture” and has a lack of social support, a consequent sense of rejection, alienation and poor self-esteem may occur” (Bhugra and Becker, 2005, p. 19). This emotional dimension deepens the metaphor of inertia in the migrant experience highlighting how migrants are excluded in dominant cultural and institutional systems.

#### 5.1.3 Reframing dominant culture through the NCMM lens

**Figure 1:**
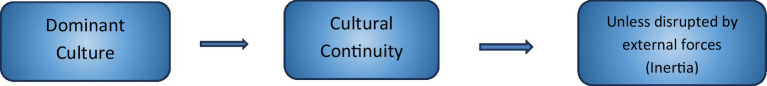
NCMM first law dynamics in migration.

Reconceptualizing Newton’s First law with Raymond Williams’ concept of dominant culture reveals how cultural inertia operates as a systemic force that resists the movement of migrant identity. This cultural inertia rooted by institutional stability and hegemonic norms make the prevailing ideologies continue which in turn make the migrant identity marginalized. This understanding of dominant culture as the inertial force allows for a more critical and a new understanding of identity politics in migratory contexts. Figure 1 illustrates the cultural inertia and dominant culture’s role in migrant identity.

### 5.2 Newton’s second law of motion and residual culture

#### 5.2.1 Residual culture as cultural mass

Newton’s Second Law states that the acceleration of an object is directly proportional to the net force acting upon it and inversely proportional to its mass (*F* = ma). In terms of cultural spheres this law helps to analyze the residual culture, which represents the beliefs, customs and norms of their roots and it does continue to exist within the evolving new cultures. In this methodological approach, mass represents the weight of the authentic traditions of the residual culture, while force represents the socio-political pressures given by the prevailing society to the migrant communities. The relationship between mass and force, as stated by Newton in his second law helps in examining identity change. That is when a cultural identity is strongly rooted carrying more “mass”, the greater external “force” is required to create a significant visible change in that identity. Through this lens NCMM emerges as a holistic tool to assess variability in identity negotiation. This method elucidates the complexities of migrant identities and provides a structured mechanism for understanding the occurrence of cultural motion within a layered system of social, historical and political influences.

#### 5.2.2 Generational shifts and uneven acceleration

**Figure 2:**
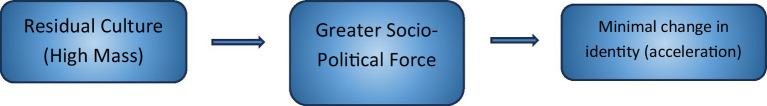
NCMM second law dynamics in migration.

In cultural terms, residual mass not only signifies the weight of tradition but also a combination of emotional attachment and the historical continuity within the migrant community. This includes kinship patterns, food habits, social and ritual practices and moral frameworks passed down intergenerationally. On the other hand, force signifies the institutional mechanisms of the host culture which aimed at reconfiguring migrant identity. These forces include cultural and language assimilation policies, economic stratification and surveillance. The metaphorical alignment of residual culture with Newton’s Second law to explain why identity transitions occur unevenly across different migrant populations. Monscheuer (2023) states, “As first-generation immigrants were socialized according to the norms in their origin countries, those originating from countries with a strong average national pride may be more likely to have a pronounced origin identity as well.” That is, the first- generation migrants, having a substantial cultural mass rooted in lived experience and social memory, exhibit the strongest resistance to external pressures, preserving the cultural lineage with greater intensity. Second-generation migrants, still shaped by these inherited norms tend to experience a partial shift that is they find themselves navigating between the values of their cultural heritage and dominant societal expectations. Third generation or the younger generational migrants are mostly embedded with the host society and their practices offering the greater alignment with the dominant culture. However, this cultural fluidity does not imply complete assimilation into dominant norms, rather it reflects the nuanced negotiation of belonging. By mapping this relationship using the NCMM researchers can get a better understanding about why certain identities persist, adapt or fragment during the course of migration. Figure 2 focuses on the Newtonian metaphor—force acting on residual cultural identity.

### 5.3 Newton’s third law of motion and emergent culture

#### 5.3.1 Emergent culture as the counter force

Newton’s Third Law states that for every action, there is an equal and opposite reaction. In the cultural context, this law metaphorically helps to correlate with the processes through which a new form of culture namely emergent culture arises as a response to dominant and residual culture. Emergent culture comprises new values, beliefs, customs, and other new forms of identity that evolve under pressures, and is often shaped by displacement, marginalization, or forced assimilation. Through the lens of the NCMM framework it is expressed that emergent culture is not formed in a spontaneous manner, but rather it is a reaction against dominant societal norms or traditional expectations. While prevailing society attempts to impose forceful assimilation, the existing culture takes the new form such as subcultures, hybrid culture, or creative resistance. These reactions do not reflect original pressures symmetrically but often exhibit equal intensity and sociocultural significance. The third law of motion thus enables scholars to trace how culture changes and evolves, and describes that identity is not passively shaped by external pressures but actively responds to them.

Within the NCMM framework emergent culture is conceptualized as the counterforce which acts against the cultural pressures of dominant culture. Younger generational migrants who are born and raised in the host country feel estranged from both the dominant cultural society and their residual culture. Their identity formation is often influenced by inherited cultural memory and trauma even without the lived experience. Hirsch (2001) describes postmemory as the intergenerational transmission of memory through stories, images and the inherited emotions. However, “postmemory need not to be strictly an identity position” (p. 10) instead it operates as the mediated relationship to the past. In NCMM, postmemory plays a significant role in forming the emergent identities by blending the force of inherited culture with the cultural pressures and the sociopolitical pressures of the host land.

#### 5.3.2 Postmemory and liminal identity formation

Migrants experience “liminality,” a condition of being in between (Van Gennep, 2013), neither belonging to the host land nor to the cultural roots of their homeland. Skjoldager-Nielsen and Edelman (2014) explain this concept of liminality as, “To define liminality in a way that reflects this tension, the term may be said to designate a transitory and precarious phase between stable states, which is marked off by conceptual, spatial and/or temporal barriers, within which individuals, groups and/or objects are set apart from society and/or the everyday” (p. 33). In this liminal space, identity becomes susceptible to reconfiguration. Zitzlsperger (2016) says that the liminal space such as hotels and railway stations acts as a symbolic threshold where cultural identities are reimagined. Emergent culture and its practices emerge as a counterforce to this dual alienation. These practices include new languages that are a blend of both native and a host land language, hybrid identity and so on. As Ashcroft et al. (2013) states, “The descendants of the diasporic movements generated by colonialism have developed their own distinctive cultures which both preserve and often extend and develop their originary cultures” (p. 62). This reflects the evolution of emergent culture through both cultural continuity and transformation, merging ancestral traditions and ideologies of the dominant society. Bhabha (1994) proposes this in-between space as the “third space.” This space is “the binary thought and essentialist identities produced by colonial knowledge” (p. 276). As Bhandari (2022) notes, “The importance of the third space does not lie in tracing the origins from which the third emerges; rather it enables other positions to evolve” (p. 173).

#### 5.3.3 Hybrid identity and the “third space” of cultural negotiation

**Figure 3:**
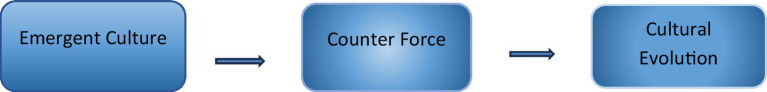
NCMM third law dynamics in migration.

Within NCMM, the third space becomes a conceptual field where cultural acceleration occurs not through assimilation or preservation but through negotiation. These spaces enable the migrant to construct emergent identities by resisting the binary frameworks. These identities carry both the ancestral memory and the ideologies conform to host norms. The emergent subject navigates this force field with hybridity and resistance offering a new form of symbolic representation. Thus, in the NCMM methodological approach of migrant studies, Newton’s third law of equal and opposite reaction not only acts as a cultural metaphor but also as a methodological principle. This method enables readers to get a different and a new insight about the emergent identity of a migrant. Emergent migrant identity is neither assimilative nor inert, rather it is a reactive and generative identity which is constantly in motion, challenging and redefining the imposed boundaries of identity and culture. Figure 3 illustrates reactionary cultural negotiation and emergent identities.

## 6 NCMM-based cultural analysis of *Pachinko*

NCMM is applied to Min Jin Lee’s *Pachinko*, to provide a sample demonstration of how cultural inertia, socio-political pressures, and the intergenerational responses shape the lived experiences of Korean migrants living in Japan.

### 6.1 Historical context of Korean migration to Japan

Korea has a 5,000 year history of its ethnic and homogenous cultural identity. It faces several territorial shifts but the current territorial boundary was set about 500 years ago. Though there is an occasional invasion of China and Japan in Korea, the cultural heredity and the real essence of the Korean ethnicity remains unchanged. “Koreans residing overseas nowadays are estimated at about 5 million, which is equivalent to 10% of the population of South Korea, or 7% of the total population in the Korean peninsula, indicating that a series of out migration waves of Koreans from the peninsula has taken place during the last one hundred years” (Kwon, 1997). Table 1 illustrates the historical data of the Koreans residing overseas in the year 1995.

**Table 1:** Koreans residing overseas, 1995.

Area/Country	Permanent	%	Temporary	%	Total	%
Asia/Pacific	2,633,115	53.3	90,805	31.3	2,723,920	52.1
China	1.926,017	39.0	14,381	5.0	1,940,398	37.1
Japan	659.323	13.4	37,488	12.9	696,811	13.3
Others	47,775	1.0	38,936	13.4	86,711	1.7
North and Latin America	1,817,238	36.8	147,512	50.8	1,964,750	37.5
United States	1,661,034	33.6	140,650	48.5	1,801,684	34.4
Canada	71.241	1.4	1,791	0.6	73,032	1.4
Latin America	84,963	1.7	5,071	1.7	90,034	1.7
Europe	486,481	9.9	40,750	14.0	527,231	10.1
CIS	459,026	9.3	2,119	0.7	461,145	8.8
Germany	17,494	0.4	11,708	4.0	29,202	0.6
Others	9,961	0.2	26,923	9.3	36,884	0.7
Middle East	340	0.0	9,016	3.1	9,356	0.2
Africa	1,171	0.0	2,145	0.7	3,316	0.1
Total	4,9,338,345	100.0	290,228	99.9	5,228,573	100.0

Adapted from Koreans in the World: Annals, Seoul: The National Unification Board, 1996, as cited in Kwon (1997).

Table 1 demonstrates the population of Koreans residing overseas in regions such as Japan, United States, Europe which together results in the vast majority of the diaspora. Since the novel *Pachinko* deals with the experiences of Korean immigrants in Japan, this historical data provides the socio-cultural backdrop. It enables an analysis of cultural shifts by using Newtonian Cultural Motion Method. By linking these historical data to the fictional narrative like *Pachinko*, the analysis gains both real migration patterns and interpretative depth of the cultural shifts through the NCMM methodological approach.

### 6.2 Application of NCMM in the novel *Pachinko*

Table 2 exemplifies the application of NCMM in the novel, *Pachinko*. In Table 2, the first column highlights the specific Newton’s law, the second column aligns these laws with Williams’ cultural theory on dominant, residual, and emergent culture, the third column presents these dynamics through the concrete examples from *Pachinko,* and the fourth column offers the NCMM interpretation, metaphorically linking the physical laws with cultural theory.

**Table 2:** NCMM application in the novel *Pachinko.*

Newton’s law of motion	Raymond Williams’ cultural concept	Application in *Pachinko*	NCMM interpretation
1. First law = (Inertia)	1. Dominant culture	1.Japanese dominance persists over generations. Koreans, though lived for decades in Japan over multiple generations, are not accepted by the Japanese and still they are treated as Zainichi, meaning a second- class citizen, a non-Japanese resident of Japan, which makes Korean immigrants feel inferior. 2.Japanese deny basic essentials to Korean immigrants. The Japanese will not rent houses to the Koreans. They are forced to live in a shabby place like *Ikaino*, the ghetto in Japan. The ghetto looks very dirty and it is not fit for humans to live in. Japanese have the dominant mindset considering “this place is fit only for pigs and Koreans” (112). Though few characters like Noa assimilates into dominant culture still he feels inferior as Japanese do not accept him. 3.Koreans find it difficult to secure employment opportunities and suitable housing, as they often face inequity in the hands of the dominant Japanese cultural norms. It significantly creates a barrier in gaining equality and acceptance in the society. 4.Even children face discrimination, in schools. Korean students are not treated equally by the Japanese students and teachers in the classrooms. They have a sense of indifference in the classrooms as the Japanese students mock them. Koreans are made to sit near the Koreans and they are not allowed to sit along with the Japanese children. They have been discriminated against as their mothers and their families raise pigs (183). The dominant outlook of the Japanese toward Koreans reflects the colonized mindset of the dominant society. Korean children are referred to as “buta,” meaning pig by Japanese children because their families raise pigs, and they are nicknamed as “garlic turd.” In the novel, a Korean schoolboy commits suicide after being repeatedly insulted and mocked by his Japanese classmates.	The dominant Japanese culture consistently excludes the integration of other cultural practices into their own culture. Despite the passage of time and generations Koreans living in Japan are still discriminated against and are still labeled as the “Zainichi” highlighting the cultural inertia of the dominant society. Japanese culture metaphorically acts as a body in motion refusing to change its direction. This resistance is seen in housing, employment and educational systems which systematically marginalize Korean immigrants. Dominant society maintains motion or resistance until it is disrupted by external forces. Japanese society in the novel *Pachinko* represents the prevailing culture and it acts as the inertia. Its strict discriminatory rules continue and resist change. Though migrants try to assimilate and adhere to (move) dominant culture, they remain the same unless external disruption occurs like any systematic changes.
2. Second law = (*F* = ma)	2. Residual culture	1.The novel *Pachinko* represents the residual culture through the way the Korean characters retain their cultural heritage despite residing in Japan. The preparation and selling of kimchi symbolizes the residual culture of Koreans living in Japan. Sunja, the protagonist’s act of selling Kimchi not only reflects her economic survival but also her determination to remain Korean. 2.Koreans resisting the Japanese dominance attitude may also be seen as the residual culture. Koreans continue to wear their traditional Korean clothes; they resist speaking in Japanese. They try to make their cultural roots alive by not assimilating into prevailing cultural norms. The Korean language has become an aid for preserving their cultural roots and holding on to Korean cultural heritage in Japanese society indicates residual culture.	Sunja’s attachment to her traditional Korean language, food, and customs highlights that the more entrenched the cultural “mass” the greater the external “force” like legal, economic crises are needed to cause cultural transitions. That is the “mass” of residual culture resists change (acceleration) unless acted upon by strong socio-political forces. Cultural change happens only if the force is strong enough, the speed of change depends on how deeply rooted the cultural identity (mass) is.
3. Third law = (Action = Reaction)	3. Emergent culture	1.A few older and younger generational Korean immigrants in *Pachinko* begin to assimilate into Japanese society by wearing Japanese-style clothes and trying to speak in Japanese. They started adapting to Japanese culture and wished to improve their Japanese accents by watching Japanese TV programs so as to be accepted by the Japanese cultural environment (138). 2.Cultural hybridity is seen as the emergent culture in the novel. “When there is thorough transformation in culture, it means that the dominant culture has been replaced with a new cultural perspective” (Baig, 2019). 3.Cultural hybridity signifies the development of a new culture that replaces the dominant culture with a different cultural perspective. The Korean characters of the novel have the hybrid identity in their names combining Korean and Japanese. One of the characters in the novel Solomon, son of Mozasu is born into an integrated, hybrid identity as he has a multiple identity of English, Japanese and Korean. In the novel *Pachinko*, the emergent culture is seen as the negotiation between Korean and Japanese culture in order to survive societal pressures.	This is the counter-force which arises as a form of tension, conflict and resistance. It challenges both dominant and residual obligations. When younger Koreans react to both Japanese dominance and Korean expectations, there arises a hybrid identity which is the blend of both Japanese and Korean culture.

Table 2 illustrates NCMM approach by integrating Newton’s Law of motion with Raymond Williams’ cultural theory on dominant, residual and emergent culture to analyze *Pachinko.* In this qualitative study, mapping dominant, residual, and emergent cultural forms onto Newton’s First, Second and Three Laws respectively, enables an exploration of the metaphorical “forces” acting upon cultural motion and transformation. This integration leads to a methodological innovation. NCMM methodological approach of the novel, *Pachinko,* reveals that the cultural identity is not only static but also a dynamic interplay of inertia, resistance and reactive motion. These forces reflect the migrant’s negotiation between historical memory, discrimination and the construction of new and hybrid identities.

## 7 Generalizability of NCMM

NCMM can be extended beyond *Pachinko* to a wide range of literary works, films, ethnographic narratives that explore migration, cultural navigation, displacement and cultural hybridity. While NCMM is demonstrated using a single novel, this methodological approach is generalizable and can also be applied to empirical studies in migration research. Researchers can use NCMM to analyze empirical data collected through interviews, surveys or ethnographic observations of migrants. The first step in applying this approach, is identifying the dominant, residual and emergent cultural components within the collected data. The directional and force-driven cultural vectors are then conceptualized in terms of the external pressures on migrants namely legal, social and economic forces. Here, the intensity of the pressure and direction is analyzed as magnitude, while the migrant’s assimilation, resistance and hybrid identities are explored in terms of direction. Finally, the observed and analyzed patterns are considered as trajectories of motion, reflecting the dynamics of cultural transformation with respect to inertia, acceleration and reaction. In this way, it is justified that NCMM is generalizable as it is applicable not only for literary analysis but also for sociological research by applying NCMM to empirical data in migration studies.

To demonstrate the generalizability and broader applicability of NCMM, a real-world migrant issue can be examined using the NCMM approach. A recent news report highlights the tension that arises in Harrisburg, Pennsylvania. The newspaper article is about the challenges faced by Bhutanese refugees in Harrisburg. “In the early 1990s, Bhutan expelled more than 100,000 Nepali-speaking Bhutanese people during a campaign of ethnic cleansing. Most fled to refugee camps in eastern Nepal, where many remained for nearly two decades” (Darjee, 2025). Initially the refugees rebuilt their lives in the host land but then everything was exploited due to political changes introduced by Trump in the U. S. which includes “deportations of many of their community members in Harrisburg,” ICE raids and so on. Migrants claim that “Trump defies everything immigrants and refugees stand for” (Darjee, 2025). Trump’s political administration creates strong socio-political pressures over the refugees.

Through NCMM analysis, the U. S legal and political norms are represented as the dominant cultural forms enforcing the deportations of the migrant community. These dominant cultural norms act as the “inertia” that persists over time. The deeply rooted cultural traditions maintained by the Bhutanese people in the host land is considered as the residual culture. This attachment toward their own tradition acts as the “cultural mass” that resists change unless acted upon by strong socio-political “forces” like government deportation orders. Emergent culture is interpreted as the adaptive mechanisms employed by the Bhutanese refugees to survive in the host land represents their “reactive motion”. This demonstration of NCMM using a real-life incident of migrants, features the need for the structured transdisciplinary methodological framework. The fact is that NCMM is applicable to divergent migrant contexts ranging from textual analysis to real-work migration scenarios.

## 8 Conclusion

Newtonian-Cultural Motion Model (NCMM), as a new methodological innovation, contributes to the migration studies and the field of cultural studies in numerous ways. Methodologically, it introduces a transdisciplinary method that integrates Newton’ s laws of motion with Raymond Williams’ dominant, residual, and emergent culture. This integration provides a structured lens for examining the efficiency of the identity formation and cultural negotiation of the migrant subjectivities. Theoretically, this study contributes to the cultural and the migrant studies by analyzing the cultural shifts of the migrants. By structuring dominant cultural norms as cultural inertia, residual culture as socio-political force and emergent culture as the counterforces, NCMM offers new insights for analyzing migrant subjectivities. Empirically, the diasporic novel *Pachinko* is chosen as the sample for demonstrating the methodology and to provide evidence to the study.

NCMM offers a new methodology for the researchers in literary studies, migration studies, and cultural theory. Its ability to bridge Newton’s law of motion and cultural analysis offers a new understanding of how identities are forged, constrained, and transformed within sociohistorical contexts. A further scope of this study lies in the adaptability of NCMM for analyzing other postcolonial or transnational narratives, offering comparative insights into the mechanics of cultural motion and resistance. The formulation of NCMM is a need as it helps researchers to trace dominant, residual and emergent cultural forces in migrant contexts. Additionally, unlike thematic interpretation, NCMM maps the cultural dynamics in terms of “motion”. It enables the researchers to trace “how” and “why” migrants undergo cultural transformations over time.

## Data Availability

The original contributions presented in the study are included in the article/supplementary material, further inquiries can be directed to the corresponding author.
